# Pain, fatigue and depression symptom cluster in survivors of prostate cancer

**DOI:** 10.1007/s00520-019-05268-0

**Published:** 2020-01-24

**Authors:** Manisha Baden, Liya Lu, Frances J. Drummond, Anna Gavin, Linda Sharp

**Affiliations:** 1 Population Health Sciences Institute, Newcastle University Centre for Cancer, Newcastle University, Level 5, Sir James Spence Institute, Royal Victoria Infirmary, Queen Victoria Road, Newcastle, NE1 4LP UK; 2 Cancer Research @ UCC, University College Cork, Cork, Ireland; 3 Northern Ireland Cancer Registry, Queen’s College Belfast, Belfast, Northern Ireland

**Keywords:** Prostate cancer, Fatigue, Pain, Depression, Symptom cluster

## Abstract

**Purpose:**

Pain, fatigue and depression are common sequelae of a cancer diagnosis. The extent to which these occur together in prostate cancer survivors is unknown. We (i) investigated prevalence of the pain-fatigue-depression symptom cluster and (ii) identified factors associated with experiencing the symptom cluster among prostate cancer survivors.

**Methods:**

Men in Ireland diagnosed with prostate cancer 2–18 years previously were identified from population-based cancer registries and sent postal questionnaires. Cancer-related pain and fatigue were measured using the EORTC QLQ-C30 and depression using the DASS-21. Cut-offs to define ‘caseness’ were pain ≥ 25, fatigue ≥ 39 and depression ≥ 10. Associations between survivor-related factors, clinical variables and specific prostate cancer physical symptoms and the symptom cluster were assessed using multivariate logistic regression.

**Results:**

A total of 3348 men participated (response rate = 54%). Twenty-four percent had clinically significant pain, 19.7% had clinically significant fatigue, and 14.4% had depression; 7.3% had all three symptoms. In multivariate analysis, factors significantly associated with the symptom cluster were living in Northern Ireland, experiencing back pain at diagnosis and being affected by incontinence, loss of sexual desire, bowel problems, gynecomastia and hot flashes post-treatment. There was a strong association between the cluster and health-related quality of life.

**Conclusions:**

The pain-fatigue-depression symptom cluster is present in 1 in 13 prostate cancer survivors. Physical after-effects of prostate cancer treatment are associated with this cluster. More attention should be paid to identifying and supporting survivors who experience multiple symptoms; this may help health-related quality of life improve among the growing population of prostate cancer survivors.

## Introduction

Among men, prostate cancer is the second most common cancer, after lung cancer, with an estimated 1.28 million new cases diagnosed worldwide in 2018 [1]. Over the past decade, prostate cancer incidence rates have risen substantially, especially in Northern and Western Europe [2]. Survival rates for prostate cancer are high (and rising) so that more men are living with this cancer than any other form of cancer [3, 4].

A range of treatment options are available for prostate cancer, including radical prostatectomy (RP), radiotherapy (external beam (EBRT) or brachytherapy (BT)), observation (active surveillance (AS) or watchful waiting (WW)) and chemotherapy. However, none of these are clearly associated with lower mortality, at least for localized disease [5]. In addition, all of the treatments pose a high risk of adverse physical effects (e.g. incontinence of urine, bowel problems and erectile dysfunction) as well as the more generalized cancer-related symptoms (e.g. pain, insomnia, fatigue), all of which can persist long-term [6, 7]. Moreover, survivors have poorer psychological wellbeing than men in the general population [8].

Cancer-related pain, fatigue and depression are recognized to co-exist in a ‘symptom cluster’ among cancer patients and survivors [9–11]. Moreover, there is some evidence that the immune/inflammation pathway provides a biological basis for the co-existence of these symptoms in a cluster [12]. However, a 2017 Expert Panel observed that symptom cluster research remains extremely limited but that the increasing focus on personalized care means that it is crucial that an understanding of individual susceptibility to symptoms and clusters of these is better understood [13].

The individual elements of the symptom cluster are common among prostate cancer survivors. Up to three-quarters of survivors may experience cancer-related fatigue; urethral pain is reported by 16% following radiation therapy; and on average, 18% of survivors have depression post-treatment [14–16]. Moreover, associations have been reported between pairs of elements of the cluster (e.g. depression and fatigue; pain and mental health) in prostate cancer survivors [8, 17]. However, as far as we are aware, no studies have investigated prevalence of the pain-fatigue-depression symptom cluster in prostate cancer or which survivors are at greatest risk of experiencing the cluster. Such information could be valuable in informing targeting of supportive care interventions.

The aim of this analysis was to (i) investigate prevalence of the pain-fatigue-depression symptom cluster among prostate cancer survivors and (ii) identify factors associated with experiencing the symptom cluster.

## Methods

### Subjects

The study setting was the island of Ireland, which comprises the Republic of Ireland (RoI) and Northern Ireland (NI; part of the UK). High-quality population-based cancer registries exist in both NI and RoI. Study methods have been described in detail elsewhere [18]. Briefly, all men diagnosed with invasive prostate cancer (ICD10 C61) between 1st January 1995 and 31st March 2010 and who were still alive on 31st March 2011 were identified through the cancer registries. A stratified random sample (*n* = 12,322, 54% of sampling frame) was selected, to ensure approximately equal numbers < 5 and ≥ 5 years post-diagnosis in both jurisdictions, and screened for eligibility by healthcare providers (HCPs), GPs in RoI and hospital nurses in NI. To be eligible, men had to be (1) alive, (2) aware of their prostate cancer diagnosis, (3) well enough to receive and complete a questionnaire (in particular, have no cognitive impairment), (4) able to understand English and (5) usually reside in RoI or NI. Following screening, 6559 survivors were considered eligible.

### Data collection

Eligible survivors were invited to complete a postal questionnaire, a copy of which is available from the authors on request. Non-responders were sent two reminders at two weekly intervals. The questionnaire collected information on sociodemographic characteristics; health at diagnosis, including presence of urinary symptoms (increased frequency, pain during urination, blood in urine), sexual symptoms (erectile dysfunction/impotence) or comorbidities (lung or heart disease, stroke, depression, diabetes, high blood pressure, bowel problems (constipation/diarrhoea), diverticular disease); mode of diagnosis (asymptomatic/prostate-specific antigen (PSA) detected, symptomatic/clinically detected, other); and treatment(s) received (RP, EBRT, androgen deprivation therapy (ADT), BT, chemotherapy, AS/WW). Men were asked to identify whether they had ever or currently experienced any of six physical after-effects following treatment (incontinence, impotence, loss of sexual desire, bowel problems (diarrhoea/constipation), gynecomastia, hot flashes/flushes or sweats). These after-effect questions, the ones present on symptoms at diagnosis, and mode of detection were developed by the authors [19, 20]. Cancer-related pain and fatigue were assessed using the European Organization for Research and Treatment of Cancer Quality-of-Life Questionnaire Version 3.0 (EORTC QLQ-C30) [21]. The global health score questions on this instrument provided a measure of health-related quality of life (HRQoL). Depression was assessed using the 21-question version of the Depression, Anxiety and Stress Scale (DASS-21) [22]. The questionnaire was pretested for face validity, acceptability and ease of completion among men with prostate cancer prior to being used. Information on clinical stage and Gleason grade at diagnosis and time since diagnosis was abstracted from cancer registry records.

The study was approved by the Ethics Committee of the Irish College of General Practitioners in the RoI and the NI Office for Research Ethics. Participants provided written informed consent.

### Statistical analysis

Of the 6559 survivors sent questionnaires, 297 were subsequently discovered to have died [18]; 3348 returned a completed questionnaire and were included in the analysis dataset. The three outcomes of interest – fatigue, pain and depression – were scored as recommended [23, 24]. For fatigue and pain, we used pro-rating to impute missing responses for subjects who answered at least half, but not all, questions in the relevant subscale; in these instances, missing responses were filled with the mean value for that subject’s responses to the questions they did answer; a fatigue score was imputed for 138 respondents (4.1%) and a pain score for 126 respondents (3.8%). The range of possible scores for fatigue and pain was 0–100 and for depression was 0–42. Initially the three variables were summarized, and Pearson correlations were computed for each pair of variables. Then, three binary variables were created classifying men according to whether or not they scored in the range for clinically significant cancer-related pain, clinically significant cancer-related fatigue and depression. The cut-offs used to define ‘caseness’ were ≥ 25 on the pain scale; ≥ 39 on the fatigue scale; and ≥ 10 on the depression scale [22, 24]. An outcome symptom cluster variable was constructed based on the presence of pain, fatigue and depression, with categories none, any one, any two and all of three symptoms. Chi-square tests were used to compare survivor characteristics by symptom cluster categories.

A multivariable logistic regression model of factors associated with presence of the symptom cluster was developed using a forward-stepwise selection approach (using a significant level < 0.05 for inclusion). In this analysis, the symptom cluster variable was collapsed into two categories: < 3 symptoms (none/any one/any two symptoms) vs all three symptoms. The candidate variables for inclusion in all models were sociodemographic characteristics (age at diagnosis and survey, country of residence, marital status, whether lived alone at diagnosis, highest level of education completed, working status, first-degree family history of prostate cancer); diagnosis characteristics (urinary or sexual symptoms at diagnosis, mode of diagnosis, comorbidities, time since diagnosis, Gleason score at diagnosis, clinical stage at diagnosis); treatment(s) received; current physical after-effects; and overall HRQoL (classified as ≥ or < median score) (Table 1). Since complete case analyses are usually biased, for the covariates, if more than 3% of men had missing data, ‘missing’ was included as a category. Goodness of fit of the final models was checked using the Hosmer and Lemeshow test. A two-sided *p* value of < 0.05 was considered to be statistically significant throughout. Analyses were performed using STATA V.15.0.

**Table 1:** Characteristics of prostate cancer survivors

	n	%
Total	3348	100.0
Sociodemographic characteristics		
Age at diagnosis (years)		
< 60	799	23.9
60–59	1631	48.7
≥ 70	918	27.4
Country of residence		
Republic of Ireland	2338	69.8
Northern Ireland	1010	30.2
Marital status		
Married/ living with a partner	2753	82.2
Other	558	16.7
Not reported	37	1.1
Live alone at diagnosis		
No	2874	85.8
Yes	424	12.7
Not reported	50	1.5
Highest level of education at diagnosis		
Tertiary	899	26.9
Secondary	1122	33.5
Primary	1187	35.4
Not reported	140	4.2
Employment status at diagnosis		
Employed/self-employed	1522	45.5
Retired	1587	47.4
Other	215	6.4
Not reported	24	0.7
First-degree family history of prostate cancer		
No	2448	73.1
Yes	791	23.6
Not reported	109	3.3
Diagnosis related		
Mode of diagnosis		
PSA-detected/asymptomatic	1860	55.6
Clinically detected/symptomatic	1148	34.3
Not reported	340	10.2
Comorbid conditions at diagnosis		
No	1476	44.1
Yes	1872	55.9
Urinating more frequently before diagnosis		
No	1387	41.4
Yes	1714	51.2
Not reported	247	7.4
Pain while urinating before diagnosis		
No	2359	70.5
Yes	256	7.6
Not reported	733	31.9
Blood in urine before diagnosis		
No	2384	71.2
Yes	219	6.5
Not reported	745	22.3
Impotence or erectile dysfunction before diagnosis		
No	2094	62.5
Yes	631	18.9
Not reported	623	18.6
Loss of interest in sex before diagnosis		
No	2179	65.1
Yes	493	14.7
Not reported	676	20.2
Back pain before diagnosis		
No	2165	64.7
Yes	506	15.1
Not reported	677	20.2
Clinical stage at diagnosis		
Stage 1	110	3.3
Stage 2	2025	60.5
Stage 3	617	18.4
Stage 4	139	4.2
Not reported/not staged	457	13.6
Gleason grade		
2 to 6	217	6.5
7 or 8	2165	64.7
8 to 10	600	17.9
Not known/not graded	366	10.9
Time since diagnosis (years)		
2–5	1614	48.2
5–10	1075	32.1
≥ 10	659	19.7
Post-treatment symptoms		
Incontinence of urine post-treatment		
No	2810	83.9
Yes	538	16.1
Impotence post-treatment		
No	1388	41.5
Yes	1960	58.5
Loss of sexual desire post-treatment		
No	1776	53.1
Yes	1572	46.9
Bowel problems post-treatment		
No	2852	85.2
Yes	496	14.8
Swelling or tenderness around nipple or upper chest area post-treatment		
No	2998	89.6
Yes	350	10.4
Sweats or hot flashes post-treatment		
No	2766	82.6
Yes	582	17.4
Treatment received^a^		
Radical prostatectomy	934	27.9
EBRT	1718	51.3
Brachytherapy	124	3.7
Hormone therapy	309	9.2
Other	170	5.1
Not reported	93	2.8
HRQoL^b^		
≥ Median	1674	50.0
< Median	1510	45.1
Not reported	164	4.9

*EBRT* external beam radiation therapy; *HRQoL* health-related quality of life

^a^Mutually exclusive hierarchical variable, based on primary treatment(s) received

^b^From EORTC QLQC30; possible range 0–100; higher score indicates better quality of life; median score 75

## Results

Of the 3348 men who responded to the questionnaire, slightly more than a quarter were 70 years of age or older at diagnosis; 35% had completed only primary level education; and almost a quarter had a first-degree family history of prostate cancer (Table 1). Presence of symptoms pre-diagnosis ranged from 7% for blood in urine to 51% for frequent urination. Nearly half of participants were 2–5 years since diagnosis. Almost one quarter had advanced stage (stage 3/4) disease at diagnosis. The most common treatments received were EBRT (51%) and RP (28%). The most common current treatment after-effects were loss of sexual desire (47%) and impotence (59%). The median HRQoL score was 75.0 (out of a possible 100).

### Prevalence of pain, fatigue and depression

Overall, at the time of the survey, 660 men (19.7%) reported clinically significant cancer-related fatigue, 802 (24.0%) had clinically significant cancer-related pain, and 481 (14.4%) had depression.

Mean scores for the three symptoms were fatigue 24.0 (sd = 24.29, lowest = 0, highest = 100); pain 15.4 (sd = 25.0, lowest = 0, highest = 100); and depression 4.5 (sd = 7.71, lowest = 0, highest = 42). Men’s scores for each pair of symptoms were strongly, and statistically significantly, correlated (pain and fatigue, rho = 0.650; depression and fatigue, rho = 0.564; depression and pain, rho = 0.454; all *p* < 0.001).

**Fig. 1:**
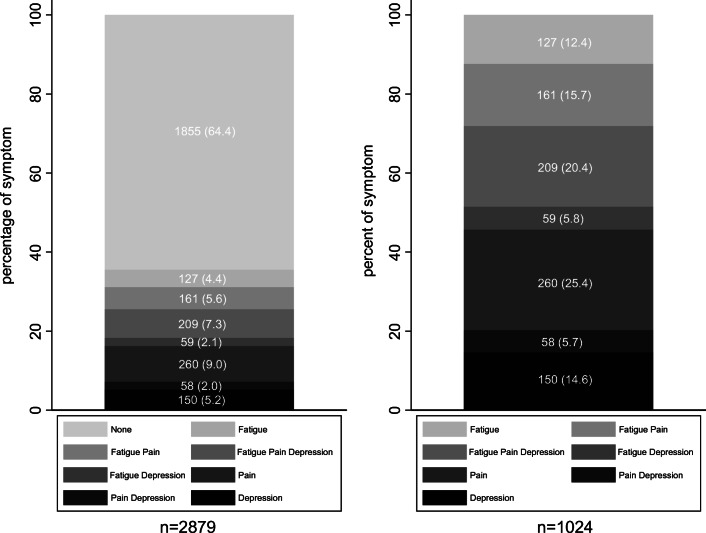
Prevalence of fatigue, pain and depression and combinations of these in prostate cancer survivors, expressed as (a) number (percentage) of survivors who completed the fatigue, pain or depression scales (*n* = 2879) and (b) number (percentage) of survivors who experienced at least one of the symptoms (*n* = 1024)

A total of 2879 (86.0%) men completed the pain, fatigue and depression scales and were therefore included in the symptom cluster analysis. Figure 1 demonstrates combinations of pain, fatigue and depression. A total of 1024 (35.6%) men experienced one or more of the symptoms in the cluster. A total of 127 men (4.4%) were affected solely by fatigue, 260 (9.0%) were affected by pain alone, and 150 (5.2%) were affected by only depression. A total of 278 men (9.7%) experienced two symptoms: fatigue and pain, 161 (5.6%); fatigue and depression, 59 (2.1%); and pain and depression, 58 (2.0%). Two hundred nine men (7.3%) had all three symptoms. Of the men who reported at least one symptom, almost half experienced two or more (two symptoms, 27.2%; all three symptoms, 20.4%).

### Factors associated with the symptom cluster

Table 2 displays the study characteristics by the four symptom cluster categories (no symptoms, one symptom, two symptoms and all three symptoms). There were significant differences in age at diagnosis, country of residence, marital status, living alone at diagnosis, highest level of education, employment status at diagnosis, mode of diagnosis, symptoms pre-diagnosis and post-treatment, clinical stage, Gleason grade, treatment received and HRQoL across the symptom cluster categories (all *p* < 0.05). Men in Northern Ireland, who lived alone, had only primary level education and, who were retired, more frequently had the pain-fatigue-depression symptom cluster. The symptom cluster was associated with having clinically detected cancer and symptoms, comorbidities, higher stage and higher Gleason grade at prostate cancer diagnosis. It was also associated with each of the six post-treatment physical symptoms assessed (urinary incontinence, impotence, loss of sexual desire, gynecomastia, sweats/hot flashes).

**Table 2:** Characteristics by symptom cluster category

	Symptom cluster category^a^				
	None (*N* = 1855)	Any one (*n* = 537)	Any two (*n* = 278)	All three (*n* = 209)	
	n (%)	n (%)	n (%)	n (%)	*p* value^b^
Sociodemographic characteristics					0.016
Age at diagnosis (years)					
< 60	458 (24.7)	133 (24.8)	73 (26.3)	57 (27.3)	
60–69	962 (51.9)	249 (46.4)	120 (43.2)	94 (45.0)	
≥ 70	435 (23.4)	155 (28.8)	85 (30.6)	58 (27.7)	
Country of residence					< 0.001
Republic of Ireland	1307 (70.5)	374 (69.7)	179 (64.4)	97 (46.4)	
Northern Ireland	548 (29.5)	163 (30.3)	99 (35.6)	112 (53.6)	
Marital status					0.015
Married/ living with a partner	1569 (84.6)	439 (81.8)	227 (81.7)	162 (77.5)	
Other	270 (14.6)	96 (17.9)	50 (18.0)	46 (22.0)	
Not reported	16	2	1	1	
Live alone at diagnosis					0.029
No	1631 (87.9)	467 (87.0)	237 (85.2)	172 (82.3)	
Yes	203 (10.9)	67 (12.5)	41 (14.8)	36 (17.2)	
Not reported	21	3	0	1	
Highest level of education at diagnosis					< 0.001
Tertiary	579 (31.2)	143 (26.6)	70 (25.2)	38 (18.2)	
Secondary	668 (36.0)	186 (34.6)	96 (34.5)	58 (27.8)	
Primary	549 (29.6)	192 (35.8)	108 (38.9)	104 (49.8)	
Not reported	59	16	4	9	
Employment status at diagnosis					< 0.001
Employed/self-employed	929 (50.1)	250 (46.6)	111 (39.9)	70 (33.5)	
Retired	855 (46.1)	250 (46.6)	129 (46.4)	107 (51.2)	
Other	66 (3.6)	34 (6.3)	37 (13.3)	31 (14.8)	
Not reported	5	3	1	1	
First-degree family history of prostate cancer					0.491
No	1371 (73.9)	386 (71.9)	213 (76.6)	156 (74.6)	
Yes	441 (23.8)	138 (25.7)	61 (21.9)	43 (20.6)	
Not reported	43	13	4	10	
Diagnosis related					
Mode of diagnosis					< 0.001
PSA-detected/asymptomatic	1157 (62.4)	287 (53.5)	125 (45.0)	81 (38.8)	
Clinically detected/symptomatic	529 (28.5)	195 (36.3)	124 (44.6)	109 (52.1)	
Not reported	169	55	29	19	
Comorbid conditions at diagnosis					< 0.001
No	939 (50.6)	199 (37.1)	96 (34.5)	54 (25.8)	
Yes	916 (49.4)	338 (62.9)	182 (65.5)	155 (74.2)	
Urinating more frequently before diagnosis					< 0.001
No	885 (47.7)	226 (42.1)	91 (32.7)	55 (26.3)	
Yes	864 (46.6)	281 (52.3)	163 (58.6)	145 (69.4)	
Not reported	106	30	24	9	
Pain while urinating before diagnosis					< 0.001
No	1400 (75.5)	382 (71.1)	186 (66.9)	141 (67.5)	
Yes	100 (5.4)	44 (8.2)	30 (10.8)	32 (15.3)	
Not reported	355	111	62	36	
Blood in urine before diagnosis					< 0.001
No	1401 (75.5)	389 (72.4)	198 (71.2)	145 (69.4)	
Yes	95 (5.1)	32 (6.0)	19 (6.8)	29 (13.9)	
Not reported	359	116	61	35	
Impotence or erectile dysfunction before diagnosis					< 0.001
No	1262 (68.0)	331 (61.6)	167 (60.1)	116 (55.5)	
Yes	298 (16.1)	112 (20.9)	65 (23.4)	61 (29.2)	
Not reported	295	94	46	32	
Loss of interest in sex before diagnosis					< 0.001
No	1323 (71.3)	343 (63.9)	171 (61.5)	124 (59.3)	
Yes	201 (10.8)	88 (16.4)	54 (19.4)	49 (23.4)	
Not reported	331	106	53	36	
Back pain before diagnosis					< 0.001
No	1347 (72.6)	335 (62.4)	166 (59.7)	104 (49.8)	
Yes	164 (8.8)	105 (19.5)	70 (25.2)	74 (35.4)	
Not reported	344	97	42	31	
Clinical stage at diagnosis					< 0.001
Stage 1	66 (3.6)	14 (2.6)	7 (2.5)	9 (4.3)	
Stage 2	1176 (63.4)	318 (59.2)	147 (52.9)	92 (44.0)	
Stage 3	336 (18.1)	108 (20.1)	55 (19.8)	56 (26.8)	
Stage 4	55 (3.0)	23 (4.3)	20 (7.2)	19 (9.1)	
Not reported/not staged	222	74	49	33	
Gleason grade					0.047
2 to 6	116 (6.3)	32 (6.0)	19 (6.8)	10 (4.8)	
7 or 8	1259 (67.9)	339 (63.1)	171 (61.5)	123 (58.9)	
8 to 10	309 (16.7)	109 (20.3)	58 (20.9)	49 (23.4)	
Not reported/not staged	171	57	30	27	
Time since diagnosis (years)					0.586
2–5	927 (50.0)	250 (46.6)	132 (47.5)	104 (49.8)	
5–10	595 (32.1)	178 (33.1)	89 (32.0)	60 (28.7)	
≥ 10	333 (18.0)	109 (20.3)	57 (20.5)	45 (21.5)	
Post-treatment symptoms					
Incontinence of urine post-treatment					< 0.001
No	1632 (88.0)	450 (83.8)	201 (72.3)	137 (65.6)	
Yes	223 (12.0)	87 (16.2)	77 (27.7)	72 (34.4)	
Impotence post-treatment					< 0.001
No	764 (41.2)	196 (36.5)	89 (32.0)	62 (29.7)	
Yes	1091 (58.8)	341 (63.5)	189 (68.0)	147 (70.3)	
Loss of sexual desire post-treatment					
No	1040 (56.1)	261 (48.6)	117 (42.1)	70 (33.5)	
Yes	815 (43.9)	276 (51.4)	161 (57.9)	139 (66.5)	
Bowel problems post-treatment					< 0.001
No	1660 (89.5)	434 (80.8)	216 (77.7)	133 (63.6)	
Yes	195 (10.5)	103 (19.2)	62 (22.3)	76 (36.4)	
Swelling or tenderness around nipple or upper chest area post-treatment					< 0.001
No	1697 (91.5)	469 (87.3)	240 (86.3)	157 (75.1)	
Yes	158 (8.5)	68 (12.7)	38 (13.7)	52 (24.9)	
Sweats or hot flashes post-treatment					< 0.001
No	1606 (86.6)	435 (81.0)	214 (77.0)	134 (64.1)	
Yes	249 (13.4)	102 (19.0)	64 (23.0)	75 (35.9)	
Treatment received					< 0.001
Radical prostatectomy	565 (30.5)	144 (26.8)	79 (28.4)	51 (24.4)	
EBRT	942 (50.8)	285 (53.1)	145 (52.2)	111 (53.1)	
Brachytherapy	91 (4.9)	18 (3.4)	5 (1.8)	3 (1.4)	
Hormone therapy	122 (6.6)	59 (11.0)	34 (12.2)	29 (13.9)	
Other	94 (5.1)	24 (4.5)	10 (3.6)	10 (4.8)	
Not reported	41	7	5	5	
HRQoL					< 0.001
≥ Median	1313 (70.8)	184 (34.3)	39 (14.0)	5 (2.4)	
< Median	528 (28.5)	343 (63.9)	235 (84.5)	201 (96.2)	
Unknown	14	10	4	3	

*EBRT* external beam radiation therapy; *HRQoL* health-related quality of life

^a^Symptom cluster included fatigue, pain and depression. Symptom cluster was categorized into none, any one of these three symptoms, any two of these three symptoms and all of these three symptoms

^b^χ^2^ test

Table 3 shows the variables that were significantly associated with presence of the symptom cluster (i.e. experiencing all three symptoms) in the multivariable model. Risk of experiencing the symptom cluster was nearly 3-fold higher in men in NI than RoI and two-fold higher in those with primary only compared to tertiary education (OR = 2.01, 95%CI 1.29–3.12). Two pre-diagnosis symptoms were significantly associated with the symptom cluster: urinating more frequently (OR = 1.53, 95% CI 1.05–2.23) and back pain (OR = 2.22, 95%CI 1.51–3.27). Four post-treatment physical symptoms were significantly related to the symptom cluster: incontinence of urine (OR = 1.91, 1.33–2.76); bowel problems (OR = 1.95, 95%CI 1.36–2.80); gynecomastia (OR = 2.06, 95%CI 1.35–3.14); and sweats or hot flashes (OR = 1.56, 95%CI 1.07–2.26). Lower HRQoL was very strongly related to the symptom cluster (OR = 44.09, 95%CI 17.88–108.73).

**Table 3:** Multivariable logistic regression analyses: associations between sociodemographic and clinical variables and the pain-fatigue-depression symptom cluster

Multivariable analyses^a^		
	OR (95% CI)	*p* value
Sociodemographic characteristics		
Country of residence		
Republic of Ireland	1.00	
Northern Ireland	2.87 (1.99–4.15)	< 0.001
Highest level of education at diagnosis		
Tertiary	1.00	
Secondary	1.55 (0.96–2.51)	0.073
Primary	2.01 (1.29–3.12)	0.002
Not reported	2.96 (1.17–7.51)	0.022
First-degree family history of prostate cancer		
No	1.00	
Yes	0.80 (0.54–1.20)	0.287
Not reported	4.15 (1.62–10.66)	0.003
Diagnosis related		
Comorbid conditions at diagnosis		
No	1.00	
Yes	1.51 (1.04–2.18)	0.029
Urinating more frequently before diagnosis		
No	1.00	
Yes	1.53 (1.05–2.23)	0.029
Not reported	1.16 (0.47–2.83)	0.752
Back pain before diagnosis		
No	1.00	
Yes	2.22 (1.51–3.27)	< 0.001
Not reported	1.08 (0.62–1.87)	0.791
Post-treatment symptoms		
Incontinence of urine post-treatment		
No	1.00	
Yes	1.91 (1.33–2.76)	0.001
Bowel problems post-treatment		
No	1.00	
Yes	1.95 (1.36–2.80)	< 0.001
Swelling or tenderness around nipple or upper chest area post-treatment		
No	1.00	
Yes	2.06 (1.35–3.14)	0.001
Sweats or hot flashes post-treatment		
No	1.00	
Yes	1.56 (1.07–2.26)	0.021
HRQoL		
≥ Median	1.00	
< Median	44.09 (17.88–108.73)	< 0.001
Unknown	36.83 (7.89–171.90)	< 0.001

*OR* odds ratio; *CI* confidence interval; *EBRT* external beam radiation therapy; *HRQoL* health-related quality of life

^a^Symptom cluster was dichotomised into less than three symptoms (none, any one or any two of these three symptoms) vs all three symptoms

Logistic model for symptom cluster, goodness-of-fit test *p* = 1.000

## Discussion

This study investigated the prevalence and factors associated with the pain-fatigue-depression symptom cluster among prostate cancer survivors. Several sociodemographic and prostate cancer specific symptoms were notably associated with this symptom cluster in univariate analyses. Once these symptoms were included in the multivariable model, many of the previously statistically significant sociodemographic and clinical factors became non-significant. The variables that remained in the model largely related to physical symptoms present pre-diagnosis (frequent urination and back pain) and after treatment (incontinence, bowel problems, gynecomastia, hot flashes). This suggests that cancer-related symptoms are more important indicators of being likely to experience this symptom cluster than sociodemographic and (majority of) clinical factors. The study also demonstrates that the three elements of the system cluster can occur alone, in pairs or all together in prostate cancer survivors, confirming previous, more general, observations [25].

### Prevalence of the symptom cluster

There is emerging (albeit inconsistent) data which suggest that inflammatory and neuroimmune markers, such as cytokines, may explain the clustering of pain, fatigue and depression in people with cancer [12]. As far as we are aware, this is the first study of this specific symptom cluster in prostate cancer survivors, and 7.3% (approximately one in every 13 survivors) experienced all three symptoms. Comparative prevalence estimates are available from relatively few studies, in part because authors have investigated and reported a variety of different combinations of symptoms as potential clusters [26]. A further complication is that, even among studies that have examined pain, fatigue and depression, different instruments were used to assess these. A recent study of 606 gastrointestinal cancer patients reported that 9.6% experienced the fatigue-pain-depression symptom cluster [27]. Higher figures have been reported in studies of patients with lung cancer (19%), which is often advanced at diagnosis, patients with advanced cancer (20%) and patients following a palliative pathway (27%) [11, 28, 29]. In terms of possible explanations for the lower observed prevalence of the symptom cluster in the current study population, this may be because the majority of survivors had localized disease at diagnosis and many had survived 10 years or more. In addition, symptoms had to be scored at a level considered clinically important to be counted.

There are many more men living with prostate cancer worldwide than with any other cancer (3.7 million men within 5 years of diagnosis alone) [30]. This indicates that, despite the lower prevalence of the symptom cluster in prostate cancer than in (some) other cancer populations, very large numbers of prostate cancer survivors worldwide may be experiencing the combination of pain, fatigue and depression (with many more living with one or two of these symptoms).

### Factors associated with the symptom cluster

As noted above, studies in which the participants had advanced cancer have generally reported higher prevalence of the symptom cluster than the current study. The observed associations between presence of physical symptoms pre-diagnosis (frequent urination and back pain) and the symptom cluster may be because these symptoms can indicate more advanced disease at diagnosis [31, 32]; thus, physical symptoms here may simply be acting as a marker of more advanced cancer. Similarly, presence of comorbid conditions has been linked with higher stage at prostate cancer diagnosis [33].

The study population was diagnosed over a long period, and there were changes in prostate cancer treatment over that time; notably radiotherapy became much more widely used, and brachytherapy was introduced [34]. We have previously reported variation in the prevalence of post-treatment symptoms among prostate cancer survivors according to primary treatment(s) received [19]. Over the time of the study, there were also differences between RoI and NI in the frequency with which different treatments were used [35]. This may help to explain the observed association between country of residence and presence of the symptom cluster in multivariable analyses.

Gynecomastia and hot flashes – two of the four post-treatment symptoms related to the symptom cluster – are side-effects of androgen deprivation therapy. Studies have previously indicated that these are associated with stigma, shame, loss of masculinity and psychological distress [36, 37]. As regards urinary incontinence, men who experience this may fear smelling or leakage of urine and find using incontinence pads embarrassing [38]; this may lead to social isolation and increased risk of depression [39]. Although bowel dysfunction has been noted to be particularly aggravating for prostate cancer survivors [40], men’s experiences of this, and its impact, have not been well investigated. It is possible that men experience discomfort due to diarrhoea or constipation and, as for urinary incontinence, may worry about leakage and embarrassment from wearing bowel incontinence pads. As well as the adverse psychological and physical effects, these physical symptoms may cause sleep disturbance [41]; sleep disorders, in turn, are linked with cancer-related fatigue [42]. Thus, the constellation of consequences of these post-treatment symptoms may explain their association with the pain-fatigue-depression symptom cluster.

Laird et al. [28] reported a strong association between the symptom cluster and worse physical functioning. We have extended these findings by showing a very strong association between lower HRQoL and the symptom cluster, which was evident after adjustment for post-treatment physical symptoms which, themselves, might be expected to impact on physical functioning. This finding has important implications.

### Implications

The current findings suggest that intervention to alleviate elements of the symptom cluster might improve survivors’ HRQoL. Both pharmacological and non-pharmacological treatments and/or interventions have been shown to be effective for the components of the cluster. Pain can be treated using resistance exercise techniques and medications [43, 44]. Mood disorders, including depression, can be treated using medication and a variety of psychotherapies, depending on the cause [45]. Physical activity also improves depression and HRQoL among survivors [46]. Cognitive-behavioural therapies, pharmacological agents and, again, physical activity (aerobic or resistance) can be effective for treating cancer-related fatigue [47, 48]. Considering this, it is possible that an intervention/treatment that is competent in addressing one symptom of the cluster (e.g. physical activity) may also alleviate another symptom (or, indeed, improve HRQoL). This provides, as noted previously by Fleishman [25], an opportunity to be imaginative in planning of survivor care and treatment strategies. However, data from the current study population indicates that supportive interventions to alleviate symptoms of prostate cancer treatment are currently not frequently used [49]. Moreover, research among clinicians indicates considerable uncertainty in how to manage concurrent symptoms in cancer patients and survivors [50], suggesting professional education initiatives may be needed.

### Strengths and limitations

Significant strengths of this study include the use of population-based sampling frames to identify survivors and the large sample size. As with any survey, there may be systematic differences between responders and non-responders. The 3348 survey respondents were younger and more often from RoI than non-respondents [18]. Among respondents, the 2879 men who answered all three sets of symptom questions were older, more often from NI, and had higher educational levels, making it unclear whether prevalence of the symptom cluster is likely to have been under- or overestimated. Measurement of the components of the symptom cluster is not straightforward. While we used validated questionnaires [21, 22] and thresholds previously shown to be associated with clinical importance [24], the EORTC QLQC30 includes only three items on fatigue and two on pain; the use of other instruments focussed specifically on cancer-related fatigue or pain, such as the EORTC QLQ-FA12 [51], may have provided richer information. We developed the questions on physical symptoms pre-diagnosis and post-treatment symptoms ourselves, and while these were pre-tested among prostate cancer survivors, information is lacking on their validity and psychometric properties; this is a limitation. The cross-sectional design means care must be taken in interpretation, particularly with regard to the direction of associations between the symptom cluster and post-treatment symptoms and HRQoL. It is possible that men with depression, fatigue and pain may assess their urinary incontinence or gynecomastia (e.g.) as worse than men who are not experiencing the symptom cluster. We have been unable to identify any population-level data on the prevalence of the combination of fatigue, pain and depression so we cannot comment on whether this is more frequent among prostate cancer survivors than the general male population.

Finally, while our focus was on the specific combination of pain, fatigue and depression among prostate cancer survivors, clusters of different combinations of symptoms among survivors of other cancers have been reported [see, e.g. 51–54]. Prostate cancer survivors are at risk of a wide range of symptoms as a result of their cancer and its treatment [6, 55] and, while it was not our intention to explore *which symptoms co-occur* among prostate cancer survivors, research to investigate this would be valuable.

## Conclusions

This study indicates that one in every 13 prostate cancer survivors experiences the pain-fatigue-depression symptom cluster at levels considered clinically important. Physical after-effects of prostate cancer treatment are noteworthy indicators of this cluster. More attention should be paid to identification and the support of survivors who experience multiple symptoms; this may help improve HRQoL among the growing population of prostate cancer survivors.
